# (*R*)-2-Cyano-*N*-(1-phenyl­eth­yl)acetamide

**DOI:** 10.1107/S1600536813008131

**Published:** 2013-04-05

**Authors:** Mohan Kumar, B. S. Madhukar, M. A. Sridhar, D. G. Bhadregowda, Kamini Kapoor, Vivek K. Gupta, Rajni Kant

**Affiliations:** a Department of Studies in Physics, Manasagangotri, University of Mysore, Mysore 570 006, India; b Department of Chemistry, Yuvarajas College, University of Mysore, Mysore 570005, India; c X-ray Crystallography Laboratory, Post-Graduate Department of Physics & Electronics, University of Jammu, Jammu Tawi 180 006, India

## Abstract

In the title compound, C_11_H_12_N_2_O, the dihedral angle between the acetamide group and the benzene ring is 68.7 (1)°. In the crystal, N—H⋯O and weak C—H⋯O hydrogen bonds link the mol­ecules into chains along the *a*-axis direction.

## Related literature

For related structures, see: Resende *et al.* (2003 ▶); Gálvez *et al.* (2010) ▶.
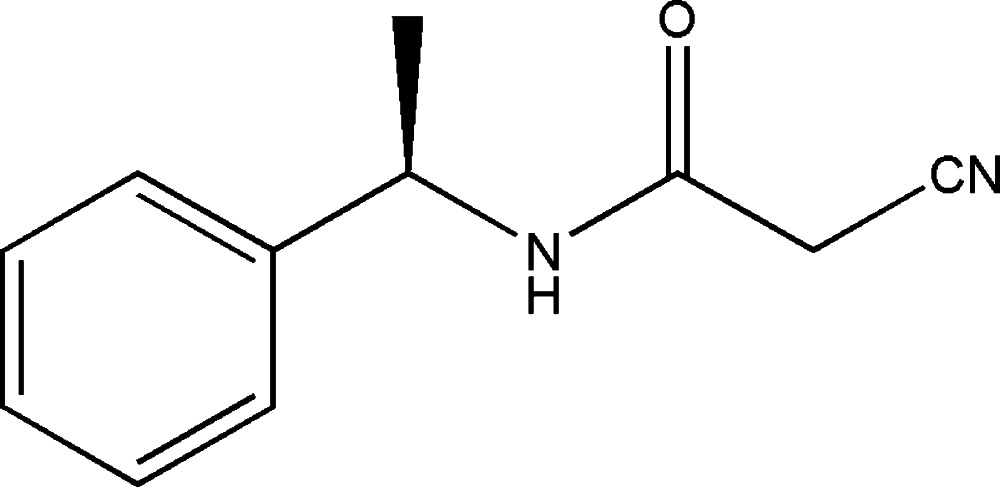


## Experimental

### 

#### Crystal data

C_11_H_12_N_2_O
*M* *_r_* = 188.23
Orthorhombic, 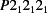

*a* = 4.7573 (1) Å
*b* = 11.1432 (3) Å
*c* = 19.3311 (5) Å
*V* = 1024.77 (4) Å^3^
*Z* = 4
Mo *K*α radiation
μ = 0.08 mm^−1^
*T* = 293 K
0.3 × 0.2 × 0.2 mm

#### Data collection

Oxford diffraction Xcalibur Sapphire3 diffractometer
Absorption correction: multi-scan (*CrysAlis PRO*; Oxford Diffraction, 2010 ▶) *T*_min_ = 0.965, *T*_max_ = 1.000
21018 measured reflections
1201 independent reflections
1097 reflections with *I* > 2σ(*I*)
*R*_int_ = 0.039

#### Refinement

*R*[*F* ^2^ > 2σ(*F* ^2^)] = 0.033
*wR*(*F* ^2^) = 0.079
*S* = 1.04
1201 reflections
128 parameters
H-atom parameters constrained
Δρ_max_ = 0.10 e Å^−3^
Δρ_min_ = −0.13 e Å^−3^

### 

Data collection: *CrysAlis PRO* (Oxford Diffraction, 2010 ▶); cell refinement: *CrysAlis PRO*; data reduction: *CrysAlis RED* (Oxford Diffraction, 2010 ▶); program(s) used to solve structure: *SHELXS97* (Sheldrick, 2008 ▶); program(s) used to refine structure: *SHELXL97* (Sheldrick, 2008 ▶); molecular graphics: *ORTEP-3* for Widows (Farrugia, 2012 ▶); software used to prepare material for publication: *PLATON* (Spek, 2009 ▶).

## Supplementary Material

Crystal structure: contains datablock(s) I, global. DOI: 10.1107/S1600536813008131/gk2561sup1.cif

Structure factors: contains datablock(s) I. DOI: 10.1107/S1600536813008131/gk2561Isup2.hkl

Supplementary material file. DOI: 10.1107/S1600536813008131/gk2561Isup3.cml

Additional supplementary materials:  crystallographic information; 3D view; checkCIF report

## Figures and Tables

**Table 1:** Hydrogen-bond geometry (Å, °)

*D*—H⋯*A*	*D*—H	H⋯*A*	*D*⋯*A*	*D*—H⋯*A*
N2—H2⋯O1^i^	0.86	2.04	2.878 (2)	165
C2—H2*A*⋯O1^i^	0.97	2.42	3.215 (2)	138

Symmetry code: (i) 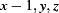.

**Fig. 1.**
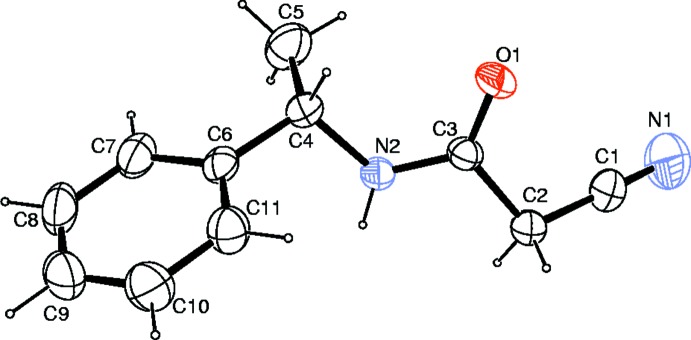
ORTEP view of the molecule with the atom-labeling scheme. The displacement ellipsoids are drawn at the 40% probability level. H atoms are shown as small spheres of arbitrary radii.

**Fig. 2.**
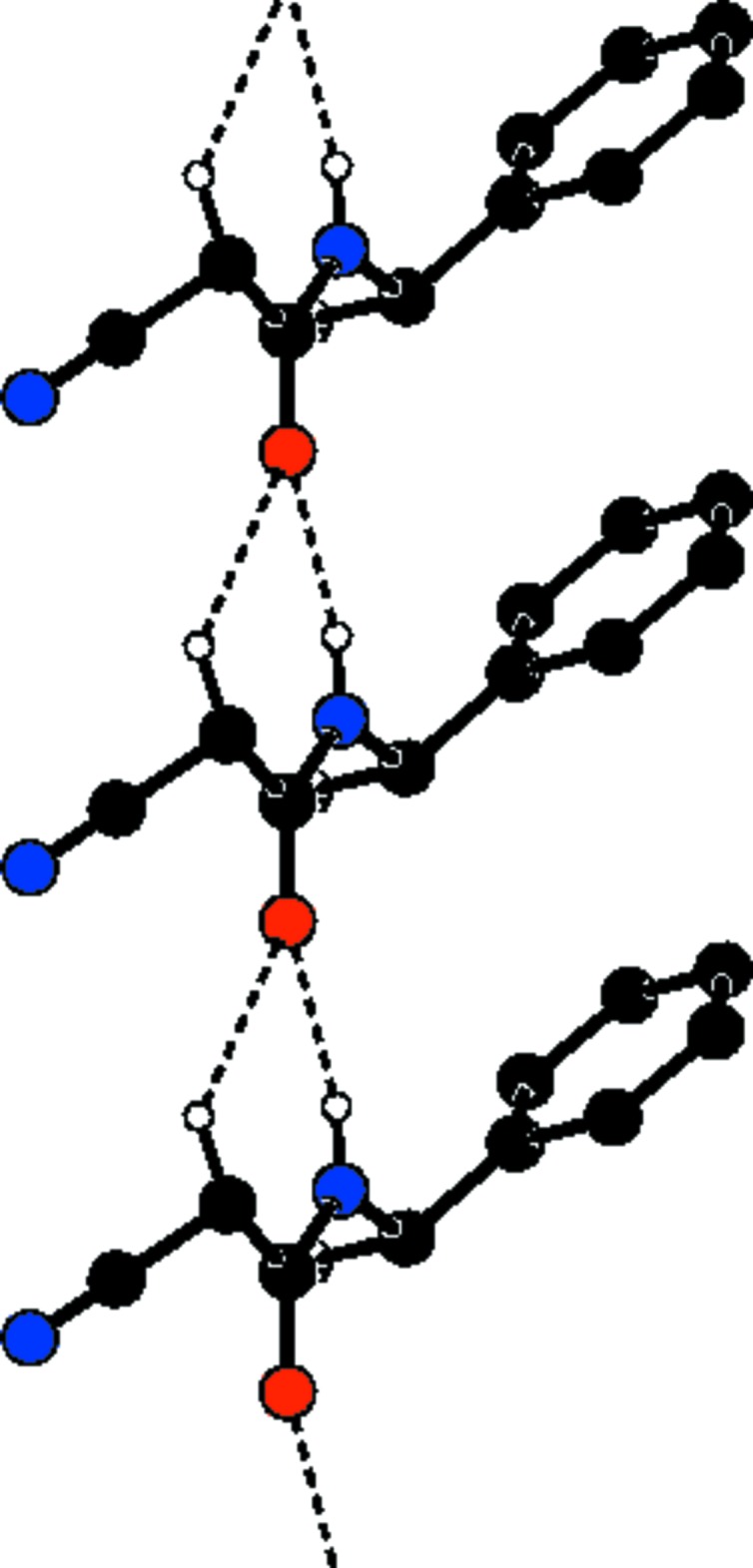
Hydrogen bonded chains of molecules via N—H···O and weak C—H···O hydrogen bonds. Only H atoms involved in hydrogen bonds are shown. Hydrogen bonds are represented by dashed lines.

**Fig. 3.**
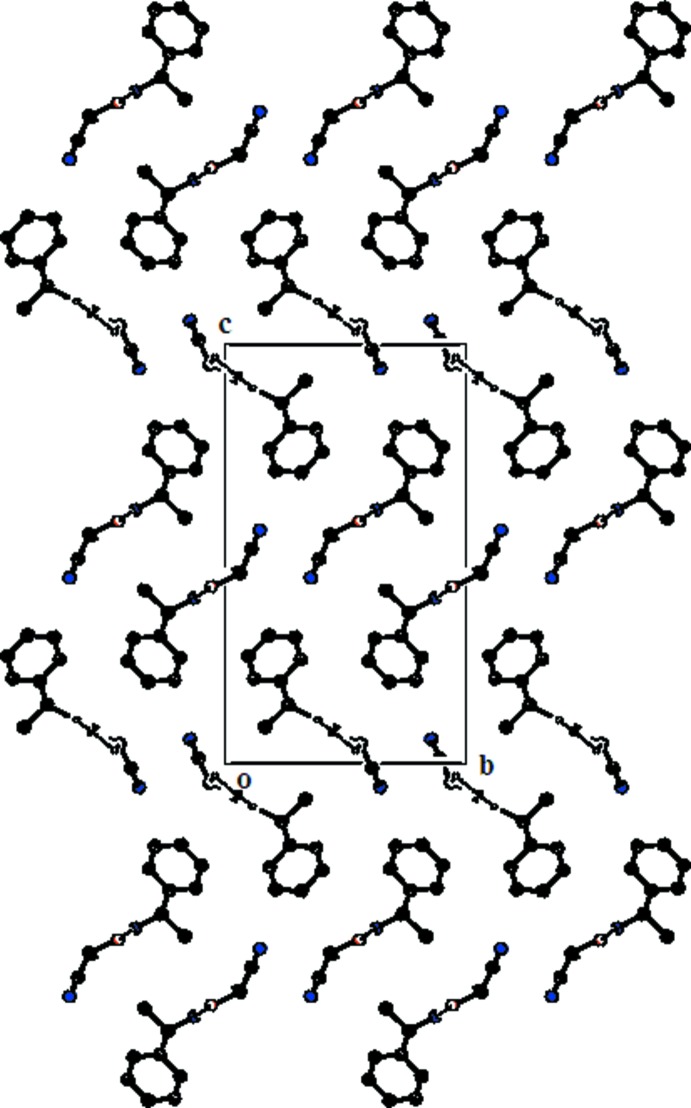
The packing arrangement of molecules viewed down the a axis. Hydrogen atoms have been omitted for clarity.

## supplementary crystallographic information

### Comment

As part of our investigations on acetamide derivatives, the title compound has been prepared and its crystal structure is presented here.Bond lengths and angles in the title compound (Fig. 1) are comparable with the similar crystal structures (Resende *et al.*, 2003; Gálvez *et al.*, 2010). The dihedral angle between the acetamide group (C2/C3/O1/N2) and the benzene ring (C6—C11) is 68.7 (1)°. N—H···O and weak C—H···O hydrogen bonds link the molecules into chains along the *a* axis (Fig.2, Table 1). In the crystal, molecules are packed into layers parallel to the bc-plane (Fig.3).

### Experimental

The reaction of methyl 2-cyanoacetate (0.1 g, 0.01 mol) and (R)-1-phenylethanamine (0.1 g, 0.01 mol) were carried out in the presence of dilute acetic acid and the reaction mixture was allowed to stir at room temperature for 6-7 h in dry dichloromethane (25 ml). The progress of the reaction was monitored by TLC. Upon completion, the solvent was removed under reduced pressure and residue was extracted with ethyl acetate. The compound was purified by successive recrystallization from methanol (yield 83%). The melting range was found to be 393-395 K.

### Refinement

All H atoms were positioned geometrically and were treated as riding on their parent C/N atoms, with C—H distances of 0.93–0.98 Å and N—H distance of 0.86 with *U*_iso_(H) = 1.2*U*_eq_(C) or 1.5*U*_eq_(methyl C). The 799 Friedel equivalents were merged. The absolute configuration could not be established from the X-ray data, but was already known from the known configuration of the starting material.

### Crystal data

**Table:**

C_11_H_12_N_2_O	*F*(000) = 400
*M**_r_* = 188.23	*D*_x_ = 1.220 Mg m^−^^3^
Orthorhombic, *P*2_1_2_1_2_1_	Mo *K*α radiation, λ = 0.71073 Å
Hall symbol: P 2ac 2ab	Cell parameters from 9923 reflections
*a* = 4.7573 (1) Å	θ = 3.6–29.1°
*b* = 11.1432 (3) Å	µ = 0.08 mm^−^^1^
*c* = 19.3311 (5) Å	*T* = 293 K
*V* = 1024.77 (4) Å^3^	Block, white
*Z* = 4	0.3 × 0.2 × 0.2 mm

### Data collection

**Table:**

Oxford diffraction Xcalibur Sapphire3 diffractometer	1201 independent reflections
Radiation source: fine-focus sealed tube	1097 reflections with *I* > 2σ(*I*)
Graphite monochromator	*R*_int_ = 0.039
Detector resolution: 16.1049 pixels mm^-1^	θ_max_ = 26.0°, θ_min_ = 3.7°
ω scans	*h* = −5→5
Absorption correction: multi-scan (*CrysAlis PRO*; Oxford Diffraction, 2010)	*k* = −13→13
*T*_min_ = 0.965, *T*_max_ = 1.000	*l* = −23→23
21018 measured reflections	

### Refinement

**Table:**

Refinement on *F*^2^	Primary atom site location: structure-invariant direct methods
Least-squares matrix: full	Secondary atom site location: difference Fourier map
*R*[*F*^2^ > 2σ(*F*^2^)] = 0.033	Hydrogen site location: inferred from neighbouring sites
*wR*(*F*^2^) = 0.079	H-atom parameters constrained
*S* = 1.04	*w* = 1/[σ^2^(*F*_o_^2^) + (0.0368*P*)^2^ + 0.1643*P*] where *P* = (*F*_o_^2^ + 2*F*_c_^2^)/3
1201 reflections	(Δ/σ)_max_ < 0.001
128 parameters	Δρ_max_ = 0.10 e Å^−^^3^
0 restraints	Δρ_min_ = −0.13 e Å^−^^3^

### Special details

**Table:**

Experimental. *CrysAlis PRO*, Oxford Diffraction Ltd., Version 1.171.34.40 (release 27–08-2010 CrysAlis171. NET) (compiled Aug 27 2010,11:50:40) Empirical absorption correction using spherical harmonics, implemented in SCALE3 ABSPACK scaling algorithm.
Geometry. All esds (except the esd in the dihedral angle between two l.s. planes) are estimated using the full covariance matrix. The cell esds are taken into account individually in the estimation of esds in distances, angles and torsion angles; correlations between esds in cell parameters are only used when they are defined by crystal symmetry. An approximate (isotropic) treatment of cell esds is used for estimating esds involving l.s. planes.
Refinement. Refinement of F^2^ against ALL reflections. The weighted R-factor wR and goodness of fit S are based on F^2^, conventional R-factors R are based on F, with F set to zero for negative F^2^. The threshold expression of F^2^ > 2sigma(F^2^) is used only for calculating R-factors(gt) etc. and is not relevant to the choice of reflections for refinement. R-factors based on F^2^ are statistically about twice as large as those based on F, and R- factors based on ALL data will be even larger.

### Fractional atomic coordinates and isotropic or equivalent isotropic displacement parameters (Å^2^)

**Table:**

	*x*	*y*	*z*	*U*_iso_*/*U*_eq_	
O1	0.4540 (3)	0.94357 (13)	0.07761 (7)	0.0500 (4)	
N1	0.3390 (6)	1.1444 (2)	−0.05721 (12)	0.0971 (8)	
N2	0.0251 (3)	0.87304 (13)	0.10524 (7)	0.0381 (4)	
H2	−0.1530	0.8858	0.1031	0.046*	
C1	0.2139 (5)	1.10928 (18)	−0.01186 (11)	0.0532 (5)	
C2	0.0562 (4)	1.06274 (17)	0.04640 (10)	0.0438 (4)	
H2A	−0.1319	1.0416	0.0313	0.053*	
H2B	0.0398	1.1246	0.0815	0.053*	
C3	0.1970 (4)	0.95288 (16)	0.07756 (8)	0.0350 (4)	
C4	0.1256 (4)	0.76346 (15)	0.13946 (9)	0.0392 (4)	
H4	0.3068	0.7823	0.1610	0.047*	
C5	0.1777 (6)	0.66621 (19)	0.08589 (11)	0.0667 (7)	
H5A	0.0050	0.6479	0.0625	0.100*	
H5B	0.2471	0.5954	0.1084	0.100*	
H5C	0.3139	0.6939	0.0529	0.100*	
C6	−0.0760 (4)	0.73086 (16)	0.19678 (8)	0.0371 (4)	
C7	−0.2066 (5)	0.62085 (18)	0.20201 (11)	0.0527 (5)	
H7	−0.1722	0.5620	0.1689	0.063*	
C8	−0.3899 (5)	0.5972 (2)	0.25659 (13)	0.0670 (7)	
H8	−0.4768	0.5226	0.2595	0.080*	
C9	−0.4434 (5)	0.6819 (2)	0.30565 (12)	0.0675 (7)	
H9	−0.5676	0.6657	0.3416	0.081*	
C10	−0.3132 (6)	0.7905 (2)	0.30156 (11)	0.0640 (6)	
H10	−0.3473	0.8486	0.3351	0.077*	
C11	−0.1309 (4)	0.81478 (19)	0.24787 (10)	0.0514 (5)	
H11	−0.0427	0.8892	0.2459	0.062*	

### Atomic displacement parameters (Å^2^)

**Table:**

	*U*^11^	*U*^22^	*U*^33^	*U*^12^	*U*^13^	*U*^23^
O1	0.0258 (6)	0.0575 (8)	0.0667 (8)	0.0020 (6)	0.0029 (6)	0.0128 (7)
N1	0.1117 (19)	0.0878 (16)	0.0917 (15)	0.0037 (16)	0.0391 (15)	0.0382 (14)
N2	0.0253 (7)	0.0399 (8)	0.0492 (8)	0.0027 (7)	−0.0020 (6)	0.0118 (7)
C1	0.0553 (12)	0.0439 (11)	0.0603 (12)	−0.0003 (10)	0.0039 (11)	0.0130 (9)
C2	0.0344 (9)	0.0414 (10)	0.0557 (10)	0.0029 (9)	0.0038 (9)	0.0109 (9)
C3	0.0289 (9)	0.0391 (10)	0.0370 (8)	0.0009 (8)	0.0001 (7)	0.0019 (8)
C4	0.0362 (10)	0.0374 (9)	0.0440 (9)	0.0049 (8)	−0.0020 (8)	0.0059 (8)
C5	0.0910 (18)	0.0519 (13)	0.0571 (12)	0.0138 (14)	0.0136 (13)	−0.0012 (10)
C6	0.0342 (9)	0.0363 (9)	0.0407 (9)	0.0025 (8)	−0.0075 (8)	0.0088 (7)
C7	0.0565 (12)	0.0385 (11)	0.0631 (12)	−0.0027 (11)	−0.0049 (11)	0.0081 (9)
C8	0.0604 (15)	0.0514 (13)	0.0892 (16)	−0.0123 (11)	0.0018 (14)	0.0289 (13)
C9	0.0589 (14)	0.0799 (17)	0.0636 (13)	0.0067 (14)	0.0136 (13)	0.0301 (13)
C10	0.0711 (15)	0.0698 (15)	0.0512 (11)	0.0076 (14)	0.0112 (12)	0.0047 (10)
C11	0.0567 (12)	0.0470 (10)	0.0504 (10)	−0.0064 (10)	0.0015 (10)	−0.0007 (9)

### Geometric parameters (Å, º)

**Table:**

O1—C3	1.227 (2)	C5—H5B	0.9600
N1—C1	1.130 (3)	C5—H5C	0.9600
N2—C3	1.322 (2)	C6—C7	1.378 (3)
N2—C4	1.469 (2)	C6—C11	1.385 (3)
N2—H2	0.8600	C7—C8	1.394 (3)
C1—C2	1.449 (3)	C7—H7	0.9300
C2—C3	1.520 (2)	C8—C9	1.362 (3)
C2—H2A	0.9700	C8—H8	0.9300
C2—H2B	0.9700	C9—C10	1.362 (4)
C4—C6	1.510 (2)	C9—H9	0.9300
C4—C5	1.519 (3)	C10—C11	1.379 (3)
C4—H4	0.9800	C10—H10	0.9300
C5—H5A	0.9600	C11—H11	0.9300
			
C3—N2—C4	122.72 (14)	C4—C5—H5C	109.5
C3—N2—H2	118.6	H5A—C5—H5C	109.5
C4—N2—H2	118.6	H5B—C5—H5C	109.5
N1—C1—C2	179.1 (3)	C7—C6—C11	117.61 (18)
C1—C2—C3	111.60 (16)	C7—C6—C4	123.66 (17)
C1—C2—H2A	109.3	C11—C6—C4	118.72 (16)
C3—C2—H2A	109.3	C6—C7—C8	120.4 (2)
C1—C2—H2B	109.3	C6—C7—H7	119.8
C3—C2—H2B	109.3	C8—C7—H7	119.8
H2A—C2—H2B	108.0	C9—C8—C7	120.9 (2)
O1—C3—N2	124.01 (18)	C9—C8—H8	119.6
O1—C3—C2	120.49 (17)	C7—C8—H8	119.6
N2—C3—C2	115.47 (15)	C8—C9—C10	119.4 (2)
N2—C4—C6	108.88 (14)	C8—C9—H9	120.3
N2—C4—C5	109.82 (15)	C10—C9—H9	120.3
C6—C4—C5	115.61 (16)	C9—C10—C11	120.2 (2)
N2—C4—H4	107.4	C9—C10—H10	119.9
C6—C4—H4	107.4	C11—C10—H10	119.9
C5—C4—H4	107.4	C10—C11—C6	121.52 (19)
C4—C5—H5A	109.5	C10—C11—H11	119.2
C4—C5—H5B	109.5	C6—C11—H11	119.2
H5A—C5—H5B	109.5		
			
C4—N2—C3—O1	0.1 (3)	C5—C4—C6—C11	179.27 (18)
C4—N2—C3—C2	−178.11 (15)	C11—C6—C7—C8	0.9 (3)
C1—C2—C3—O1	33.0 (3)	C4—C6—C7—C8	179.84 (18)
C1—C2—C3—N2	−148.74 (17)	C6—C7—C8—C9	0.0 (3)
C3—N2—C4—C6	148.29 (16)	C7—C8—C9—C10	−0.7 (4)
C3—N2—C4—C5	−84.2 (2)	C8—C9—C10—C11	0.6 (4)
N2—C4—C6—C7	124.55 (18)	C9—C10—C11—C6	0.4 (3)
C5—C4—C6—C7	0.4 (3)	C7—C6—C11—C10	−1.1 (3)
N2—C4—C6—C11	−56.6 (2)	C4—C6—C11—C10	179.93 (18)

### Hydrogen-bond geometry (Å, º)

**Table:**

*D*—H···*A*	*D*—H	H···*A*	*D*···*A*	*D*—H···*A*
N2—H2···O1^i^	0.86	2.04	2.878 (2)	165
C2—H2*A*···O1^i^	0.97	2.42	3.215 (2)	138

Symmetry code: (i) *x*−1, *y*, *z*.
